# Antipruritic Effect of Ethyl Acetate Extract from *Fructus cnidii* in Mice with 2,4-Dinitrofluorobenzene-Induced Atopic Dermatitis

**DOI:** 10.1155/2020/6981386

**Published:** 2020-05-06

**Authors:** Xi Chen, Chan Zhu, Yingge Zhang, Niuniu Yang, Hao Shi, Weiwei Yang, Yan Yang, Jianqiang Liang, Liuzhi Chen, Xueying Zeng, Rijin Cai, Guanyi Wu, Zongxiang Tang

**Affiliations:** ^1^College of Pharmacology, Guangxi Medical University, 22 ShuangYong Road, Nanning 530200, China; ^2^School of Medicine & Holistic Integrative Medicine, Nanjing University of Chinese Medicine, 138 XianLin Road, Nanjing 210023, China; ^3^College of Basic Medicine, Yangzhou University, 88 Daxue South Road, Yangzhou 225009, China; ^4^Department of Clinical Medicine, Kangda College of Nanjing Medical University, 88 ChunHui Road, Lianyungang 222000, China; ^5^College of Basic Medicine, Guangxi University of Chinese Medicine, 13 WuHe Road, Nanning 530299, China

## Abstract

Atopic dermatitis (AD) is a common inflammatory skin disease characterized by intense pruritus and skin lesions. The exact cause of AD is not yet known and the available therapeutic strategies for AD are limited. *Fructus cnidii* is commonly used in traditional Chinese medicine as an herb for treating chronic itch. However, the mechanism underlying the antipruritic effects of *Fructus cnidii* is not well understood. In the present study, we investigated the antipruritic effect of locally administered ethyl acetate extract from *Fructus cnidii* (EAEFC) to 2,4-dinitrofluorobenzene- (DNFB-) induced AD in a mouse model. The scratching behavior, skin thickness, dermatitis score, weight, blood immunoglobulin E (IgE) level, and itch-related cytokine levels were subsequently monitored and evaluated. Results showed that EAEFC treatment attenuated the DNFB-induced AD-like symptoms by alleviating the skin lesions and decreasing the dermatitis score. Hematoxylin and eosin (H&E) and toluidine blue (TB) staining analyses demonstrated that EAEFC mitigated the DNFB-induced increase in skin thickness and prevented the infiltration of mast cells. Behavioral tests showed that EAEFC decreased the DNFB-induced acute and chronic scratching behaviors. Furthermore, EAEFC reduced the levels of itch-related cytokines, such as thymic stromal lymphopoietin (TSLP), interleukin- (IL-) 17, IL-33, and IL-31, and the DNFB-induced boost in serum IgE. Collectively, these results suggest that EAEFC is a potential therapeutic candidate for the treatment of chronic itch in AD.

## 1. Introduction

Atopic dermatitis (AD), one of the most common refractory and chronic inflammatory skin disease, is generally characterized by pruritus, epidermal barrier damage, eczematous skin, papule, seropapule, vesicle, squames, crusts, and abnormal immunological responses [1, 2]. Pruritus is the major symptom of AD, with an expected prevalence rate between 2% and 5% [3, 4]. A patient's quality of life becomes severely affected due to chronic pruritus [4–6]. AD patients often suffer from insomnia, anxiety, depression, and other emotional disorders [4, 7, 8]. Several studies have been focused on understanding the symptoms and mechanisms of AD; however, these have not yet been fully elucidated, which has limited the development of novel therapeutic strategies.

Itch is an unpleasant sensation that induces a desire to scratch, which may be acute or chronic (lasts for 6 weeks or more) [9, 10]. Serious chronic itch is the primary and most problematic feature of AD, with a reported prevalence ranging from 87% to 100% [11]. The complex interaction of various distinct mediators, including cytokines, neuropeptides, and endogenous secreted factors, can induce pruritus [11]. Certain cytokines, such as interleukin (IL-) 17, IL-33, IL-31, and thymic stromal lymphopoietin (TSLP), play an important role in the development of pruritus [11–13]. IL-4 and IL-13 are type 2 helper T (Th2) cells that directly activate the sensory neurons and itch-sensory pathways for enhancing neuronal responsiveness to multiple pruritogens; however, this does not directly induce scratching [12]. IL-17 produced by type 17 T helper (Th17) cells, which is a subset of CD4^+^ T helper cells, is significantly elevated in the skin and associated with IL-31, a pruritus cytokine in canine AD [14, 15]. IL-33 belongs to the IL-1 family of cytokines and promotes Th2 immune responses [16]. Liu et al. reported that exogenous IL-33 either exacerbated the itch-related scratching behaviors in mice with urushiol-induced allergic contact dermatitis or directly induced the skin scratching behaviors 4 h after injection [17]. Moreover, IL-33 can activate the dorsal root ganglion neurons and induce calcium influx, which are involved in the chronic itch caused by poison ivy contact allergy [17]. TSLP, which is highly produced in cutaneous epithelial cells and keratinocytes, is also regarded as a pruritogen that can induce scratching behaviors in AD. The TSLP released from keratinocytes activated the primary afferent neurons directly mediated by the action on TSLP receptors and opens the ion channel, TRPA1, but not TRPV1 [18]. Studies also revealed that the immune cells, activated by TSLP and inflammatory mediators, secreted other inflammatory mediators that can activate sensory neurons and induce itch [18, 19]. One notable research reported that IL-31, belonging to the IL-6 family of cytokines, is generated by Th2 cells and has significantly increased levels in AD [20]. IL-31 is a known endogenous pruritogen that plays an important role in pruritus development by promoting the release of inflammatory cytokines and the growth of the sensory nerve [21, 22]. IL-31 injected subcutaneously into a dog, monkey, or mouse model can directly elicit scratching by binding with a heterodimeric receptor, which is composed of IL-31 receptor *α* (IL-31RA) and oncostatin M receptor *β* (OSM) [21, 23].

**Figure 1:**
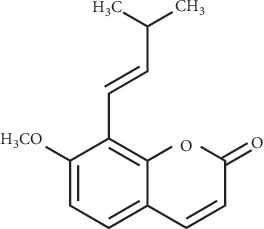
Chemical structure of osthole.

*Fructus cnidii*, the dried fruit of *Cnidium monnieri* (L.) Cusson, is an herbal medicine called “shechuangzi” in China that was reported to have antiallergic, anti-inflammatory, and antipruritus properties [24, 25]. It is mainly used in traditional Chinese medicine as a special treatment for AD, asthma, psoriasis, urticaria, ringworm, and osteoporosis [24, 25]. Matsuda et al. reported that compound 48/80 (a condensation product of N-methyl-p-methoxyphenethylamine with formaldehyde) induced scratching behavior was inhibited by the ethanol extract of *Fructus cnidii* [26]. Our previous research has revealed that osthole (7-methoxy-8-isopentenyloxycoumarin), which is the main component of *Fructus cnidii*, can prevent histamine-dependent acute itch by inhibiting the TRPV1 nonselective ionic channel (Figure 1) [27]. The Kewei Wang research group reported that osthole was involved in reducing the scratching behaviors due to acute itch and AD in mice through the inhibition of TRPV3 [28, 29]. Furthermore, osthole has an antipruritic effect in allergic animal models and alleviates AD by directly downregulating TSLP production in keratinocytes [30, 31]. Contrastingly, the antipruritic activity of *Fructus cnidii*, specifically the mechanism associated with itch-related cytokines that may potentially relieve the chronic itch in AD, is not clearly understood.

In the present study, we aimed to demonstrate the antipruritic effect of ethyl acetate extract from *Fructus cnidii* (EAEFC) in AD mouse model. Our results showed that EAEFC alleviated the AD skin lesions, epidermal hyperplasia, and mast cell infiltration. In addition, EAEFC attenuated the chronic scratching behaviors and reduced the levels of itch-related cytokines.

## 2. Materials and Methods

### 2.1. Preparation of EAEFC

The dried *Fructus cnidii* seeds were purchased from Nanning Medicine Market (Guangxi, China) and identified by Prof. Dan Zhu from the Guangxi Medical University. *Fructus cnidii* powder (1,000 g) was placed in a round-bottom flask, 1,000 mL 80% ethanol was added, and the mixture was boiled for 1 h. This extraction procedure was repeated three times. The mixtures were collected and filtered to obtain the solution. The ethanol was removed by rotary evaporation, while the remaining water was removed by incubating the solution on a 75°C water bath. The residual oily substance, which was the alcohol extract of *Fructus cnidii*, was completely dissolved in water. The aqueous solution was extracted three times with ethyl acetate (1 : 3, ethyl acetate: water solution, v/v). The organic phase was collected and the ethyl acetate was removed by rotary evaporation. The obtained EAEFC was sealed and stored at 4°C until use.

### 2.2. High-Performance Liquid Chromatography (HPLC) Analysis

HPLC analysis was conducted using Agilent 1100 Series HPLC System (Agilent Technologies Inc., Palo Alto, CA, USA). EAEFC (1.5 g) was dissolved in 15 mL 80% ethanol and filtered. This procedure was repeated three times. The filtrate was collected and diluted to 50 mL using 80% ethanol. The analysis was performed at 30°C on a C18 column (250 mm × 4.6 mm, 5 *μ*m) using 10 *μ*L sample and 75 : 25 methanol: water, with a flow rate of 0.8 mL·min^−1^ and absorbance at 322 nm.

### 2.3. Experimental Animals and Groups

A total of 40 six-week-old male C57BL/6 mice were used as the AD model. The animals were maintained in a temperature-controlled room at 22 ± 2°C under a 12-h light/12-h dark cycle and with free access to food and water. All experiments were carried out in accordance to the guidelines and regulations approved by the Institutional Animal Care and Use Committee of the Nanjing University of Chinese Medicine. The mice were randomly divided into five groups: (1) control: untreated, (2) model group: 2,4-dinitrofluorobenzene- (DNFB-) treated, (3) low-dose group: DNFB-treated + EAEFC-treated (50 mg·kg^−1^), (4) high-dose group: DNFB-treated + EAEFC-treated (200 mg·kg^−1^), and (5) positive control group: DNFB-treated + tacrolimus-treated. The EAEFC dosages were chosen based on previous studies [26, 32].

### 2.4. Drugs and Compounds

DNFB and acetone were purchased from Sigma-Aldrich (St. Louis, MO, USA). To induce AD-like symptoms and skin lesions, DNFB was applied to the dorsal skin and subsequently treated with EAEFC and tacrolimus. DNFB (0.2% or 0.5%) was dissolved in a 4 : 1 acetone: olive oil (v/v) mixture [33, 34]. The DNFB solution should be freshly prepared before use. EAEFC (50 mg·kg^−1^ or 200 mg·kg^−1^) was dissolved in the vehicle solution (3 : 1 acetone: distilled water, v/v) [35]. The equivalent volume of vehicle solution (without EAEFC) was applied to the control and DNFB-treated groups. Osthole was purchased from Shanghai Yuanye Biotechnology Co., Ltd. (Shanghai, China). The DNFB and EAEFC solutions (100 *μ*L each) were applied to the neck. Tacrolimus ointment 0.1% (Protopic®, Wangsheng Pharma, Hangzhou, China) was smeared on the skin of mice at a dose of 100 mg·day^−1^ per mouse.

### 2.5. AD Model Treatment

**Figure 2:**
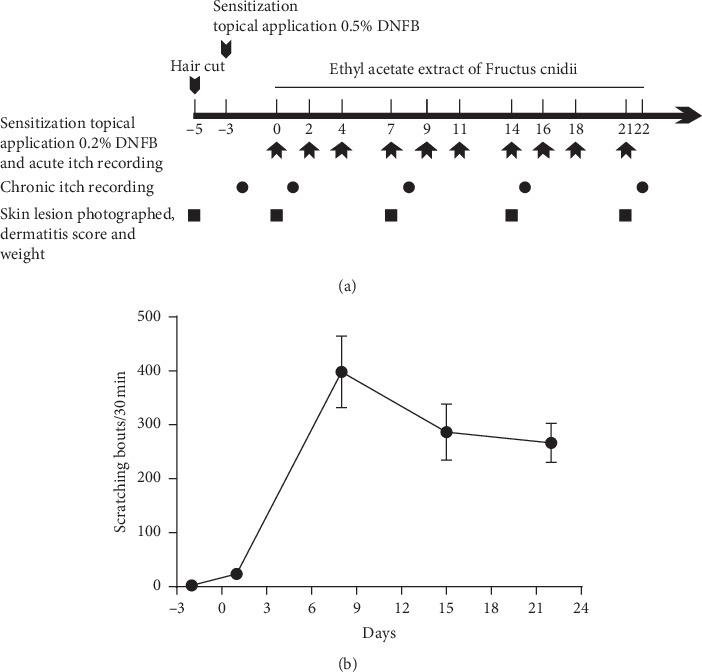
Experimental scheme and chronic itch model of AD. (a) Experimental scheme: dorsal neck hair removal on day -5. The 0.5% DNFB was applied to the abdomen (day -3) and the 0.2% DNFB was applied to the dorsal neck (day 0). The animals were sensitized with 0.2% DNFB thrice a week for three weeks (days 0 to 21). EAEFC or tacrolimus was topically applied twice a day for three weeks (days 0 to 21). Chronic scratching behavior was recorded five times on days −2, 1, 8, 15, and 22. (b) In the chronic itch model of AD, the scratching bouts increased after the first week, which can also be seen in Figure 8.

First, the dorsal neck skin (3 cm × 3 cm) and abdomen skin (4 cm × 4 cm) of six-week-old mice were shaved using an electric hair clipper five days before the experiments (day-5). Second, 0.5% DNFB (100 *μ*L) was applied to the abdomen on day-3 to sensitize the skin, while 0.2% DNFB (100 *μ*L) was applied to the neck on day 0 (Figure 2(a)). Finally, the dorsal neck challenge was maintained thrice a week for three weeks with 0.2% DNFB (Figure 2(a)). EAEFC (50 mg·kg^−1^ and 200 mg·kg^−1^, 100 *μ*L each) and tacrolimus (100 mg) were applied to the neck starting from the first day of the challenge. EAEFC and tacrolimus were administered twice a day at 10 AM and 4 PM until the end of the experiment. The main experimental protocol is described in Figure 2(a).

### 2.6. Skin Lesions, Dermatitis Score, and Weight Assessment

The method to assess the skin lesions and dermatitis score was described by Kim et al. (2014) [36]. On day 22, the mice were anesthetized with isoflurane and the skin lesions were photographed using a Sony HDR-PJ790E digital video camera (Sony Corporation, Tokyo, Japan). Based on the criteria reported in literature, the severity of dermatitis was measured on days −5, 0, 7, 14, and 21. Four symptoms, specifically, erythema/hemorrhage, edema, excoriation/erosion, and scaling/dryness, were classified into four levels and scored as follows: 0 (none), 1 (mild), 2 (moderate), and 3 (severe). All mice were scored before receiving DNFB treatment. The mice were also weighed on days −5, 0, 7, 14, and 21 using an electronic scale.

### 2.7. Analysis of Scratching Behavior

The method described by Kuraishi et al. was used to evaluate the scratching behavior in the experimental animals. The mice were maintained in a transparent plastic box (4.5 in × 4.5 in × 7 in) for approximately 30 min to prevent acclimatization before each experiment. Next, the scratching behaviors were monitored and recorded for 30 min. One scratch response was defined as the lifting of the hind limb towards the injection site. Chronic scratching was recorded using the same digital video (Sony) on days −2, 1, 8, 15, and 22, which was the day after DNFB challenge. Acute scratching in three mice from each group was recorded on the day of the DNFB challenge immediately after application.

### 2.8. Histological Analysis

The shaved part of the neck skin was excised on the last day of the experiment. The skin samples were fixed with 4% paraformaldehyde for 2-3 days, precipitated with 30% sucrose for 24 h, and embedded in frozen OCT embedding compound (Sakura Finetek USA, Inc., Torrance, CA, USA). Next, the skins were sliced into 5 *μ*m sections using a CM1950 Cryostat (Leica Biosystems Nussloch GmbH, Wetzlar, Germany). The skin sections were stained with hematoxylin and eosin (H&E) and toluidine blue (TB) for the analyzing the epidermal thickness and the number of mast cells, respectively. The specimens were observed under 400x magnification, with five random fields of vision per slice, using the Axio Zoom V16 optical microscope (Carl Zeiss AG, Oberkochen, Germany).

### 2.9. Measurement of IgE and Itch-Related Cytokines

Blood samples were collected on day 22 of the experiment. The serum was centrifuged for 15 min at 4500 ×g at 4°C and stored at −80°C until use. The serum IgE, IL-17, IL-33, IL-31, and TSLP levels were measured using an enzyme-linked immunosorbent assay (ELISA) kit (Bioss, Beijing, China) following the manufacturer's instructions. The reaction product was measured colorimetrically at 450 nm using a microplate reader (Molecular Devices, LLC, Sunnyvale, CA, USA). The raw fluorescence data were analyzed using a 4-parameter logistic method.

### 2.10. Statistical Analysis

All data values were expressed as mean ± S.E.M. The mean values among groups were analyzed and compared by one-way analysis of variance (ANOVA), followed by Tukey's multiple comparison test, using GraphPad Prism version 8.0 software (GraphPad Software Inc., San Diego, CA, USA). Differences were considered statistically significant at ^*∗*^*P* < 0.05, ^*∗∗*^*P* < 0.01, and ^*∗∗∗*^*P* < 0.001.

## 3. Results and Discussion

### 3.1. HPLC Analysis of EAEFC

**Figure 3:**
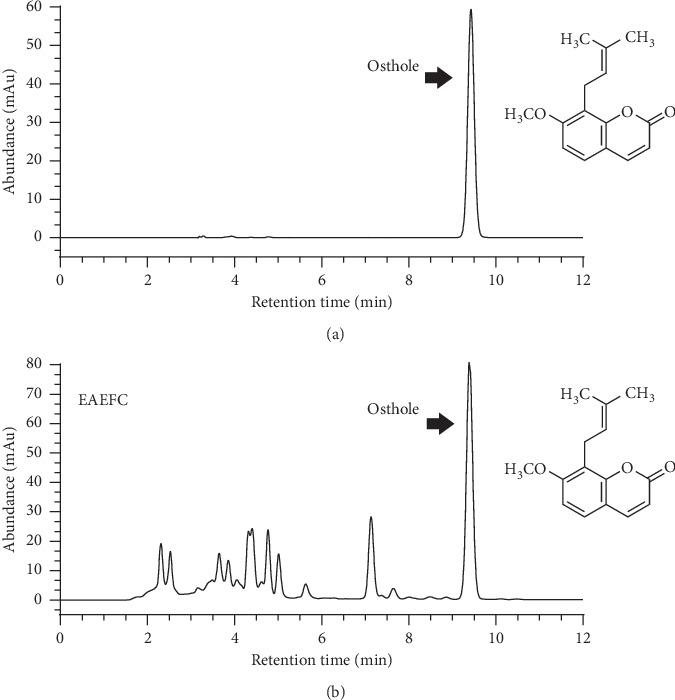
Representative HPLC chromatograms. (a) Osthole standard. (b) EAEFC sample.

Osthole was identified as the main compound in EAEFC through HPLC analysis (Figure 3), which compared the retention time of the sample with the standard osthole. The peak of EAEFC at 9.39 min in the chromatogram was almost similar to the peak of the standard osthole at 9.43 min (Figure 3), which suggests that the compound in EAEFC may be osthole. Osthole had the highest peak area percentage (30.5%) among all the EAEFC contents.

### 3.2. Characteristics of DNFB-Induced AD-Like Symptoms in C57BL/6 Mice

**Figure 4:**
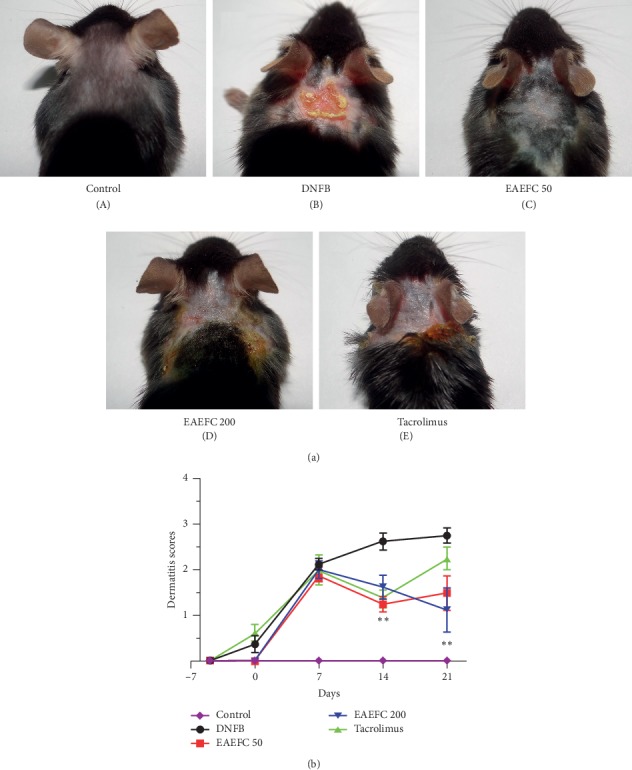
Effect of EAEFC treatment on the skin lesions and dermatitis score of C57BL/6 mice with DNFB-induced AD. (a) Images of skin lesions were taken on day 22. (b) The dermatitis scores were evaluated on days −5, 0, 7, 14, and 21. ^*∗∗*^: Significant difference between the EAEFC200 and DNFB-treated groups at *P* < 0.01. Data are presented as the mean ± SEM (*N* = 8).

To investigate the antipruritic effect of EAEFC on the AD animal model, the mice were sensitized with 0.5% DNFB and challenged with 0.2% DNFB. Results showed that several mice immediately displayed scratching behavior (2 ± 1, *N* = 8) after day 1. On day 8, the maximum scratching bouts were 398 ± 66 (*N* = 8) (Figure 2(b)). The scratching response decreased on days 15 (286 ± 52, *N* = 8) and 22 (267 ± 36, *N* = 8); however, the number of scratching bouts remained at a high level (Figure 2(b)). In contrast, the number of scratching bouts in the control group did not change over time and the total number of scratching bouts recorded was less than 10. The DNFB group exhibited severe skin lesions, erythema, erosion, and ulceration (Figure 4(a)) and a dermatitis score relatively higher than the control group, which further increased after day 7. Moreover, the dermatitis score of the DNFB group (2.8 ± 0.2, *N* = 8) was significantly different from the control group on day 21 (2.8 ± 0.2 vs. 0 ± 0, *N* = 8, ^*∗∗∗*^*P* < 0.001). These results indicate that AD was successfully induced using DNFB, which was consistent with previous studies.

### 3.3. EAEFC Alleviated the DNFB-Induced Skin Lesions in C57BL/6 Mice

To investigate whether EAEFC can alleviate the DNFB-induced AD-like skin lesions in C57BL/6 mice, images of skin lesions were photographed and scored. EAEFC and tacrolimus alleviated the skin lesions in mice starting from day 14. On day 21, the 50 mg·kg^−1^ EAEFC (EAEFC50) group had slightly alleviated skin lesions (Figure 4(a)). The dermatitis score of the EAEFC50 group significantly decreased compared to the DNFB-treated group (1.5 ± 0.4 vs. 2.8 ± 0.2, *N* = 8, ^*∗*^*P* < 0.05) (Figure 4(b)). Furthermore, the 200 mg·kg^−1^ EAEFC (EAEFC200) group had better recovered skin, lessened swelling, and ulceration, which correspond to significantly alleviated skin lesions. The EAEFC200 group had a lower dermatitis score compared to the DNFB-treated group (1.1 ± 0.5 vs. 2.8 ± 0.2, *N* = 8, ^*∗∗*^*P* < 0.01) (Figure 4(b)). The EAEFC200 treatment on the mice model did not result in any visible side effects. These results indicate that EAEFC effectively alleviated the skin lesions of DNFB-induced in C57BL/6 mice.

### 3.4. Effect of EAEFC on the Weight of C57BL/6 Mice with DNFB-Induced AD-Like Symptoms

**Figure 5:**
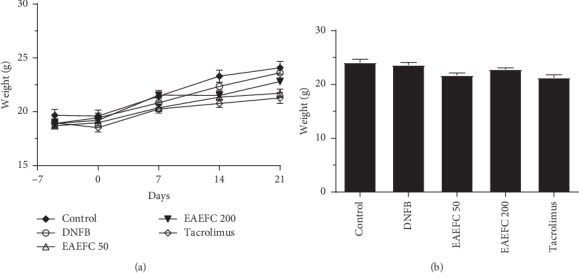
Effect of EAEFC treatment on the weight of C57BL/6 mice with DNFB-induced AD. (a) The weight of mice from each group was measured on days −5, 0, 7, 14, and 21. (b) The weight of mice from each group on day 22. Data are presented as the mean ± SEM (*N* = 8).

To investigate the effect of EAEFC on the weight of mice with DNFB-induced AD, the weight of mice were recorded on days −5, 0, 7, 14, and 21. Results showed that the mice in each group had gained weight over time; however, no significant difference between the DNFB-treated and other groups was observed (Figure 5(a)). On day 21, there was still no significant difference between the EAEFC200 and DNFB-treated groups (23.6 ± 0.5 *vs* 22.8 ± 0.3, *N* = 8, *P*=0.175) (Figure 5(b)). In contrast, there were significant differences between the control and EAEFC50 groups (24.1 ± 0.6 vs. 21.7 ± 0.4, *N* = 8, ^*∗∗*^*P* < 0.01) and the control and tacrolimus-treated groups (24.1 ± 0.6 vs. 21.3 ± 0.5, *N* = 8, ^*∗∗*^*P* < 0.01). These results indicate that the frequent scratching might have dampened the appetite and consequently inhibited the weight gain in mice.

### 3.5. EAEFC Alleviated the DNFB-Induced Epidermal Hyperplasia and Inflammatory Cell Infiltration in C57BL/6 Mice

**Figure 6:**
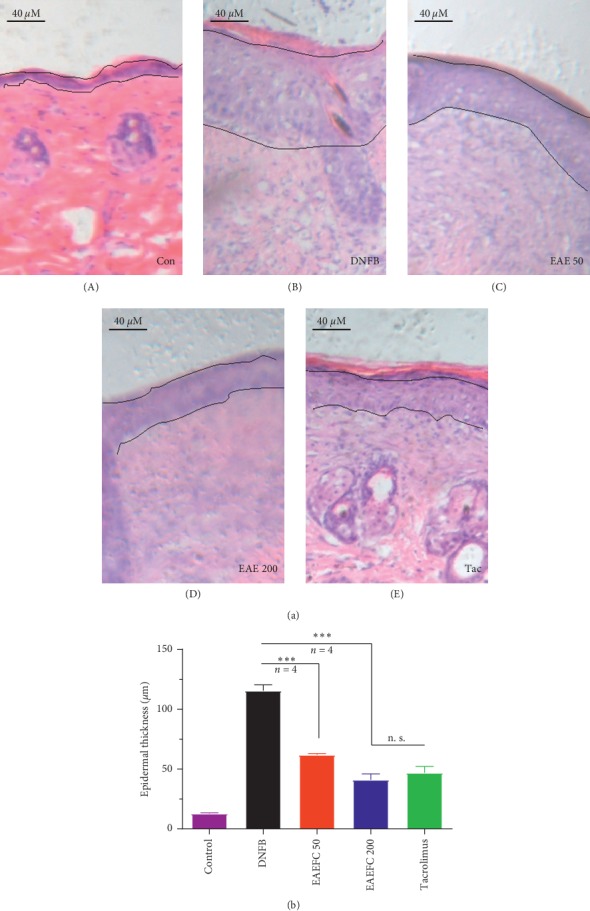
Effect of EAEFC treatment on the DNFB-induced increase in epidermal thickness of C57BL/6 mice. (a) H&E-stained skin on day 22. The dotted line indicates the epidermis and hypertrophy in the (A) control group, (B) DNFB-treated group, (C) EAEFC50 group, (D) EAEFC200 group, and (E) tacrolimus-treated group. (b) Data are presented as the mean ± SEM (*N* = 4). ^*∗∗∗*^: Significant difference between the EAEFC50 and DNFB-treated groups and between the EAEFC200 and DNFB-treated groups at *P* < 0.001. n s.: No significant difference between the EAEFC200 and tacrolimus-treated groups.

**Figure 7:**
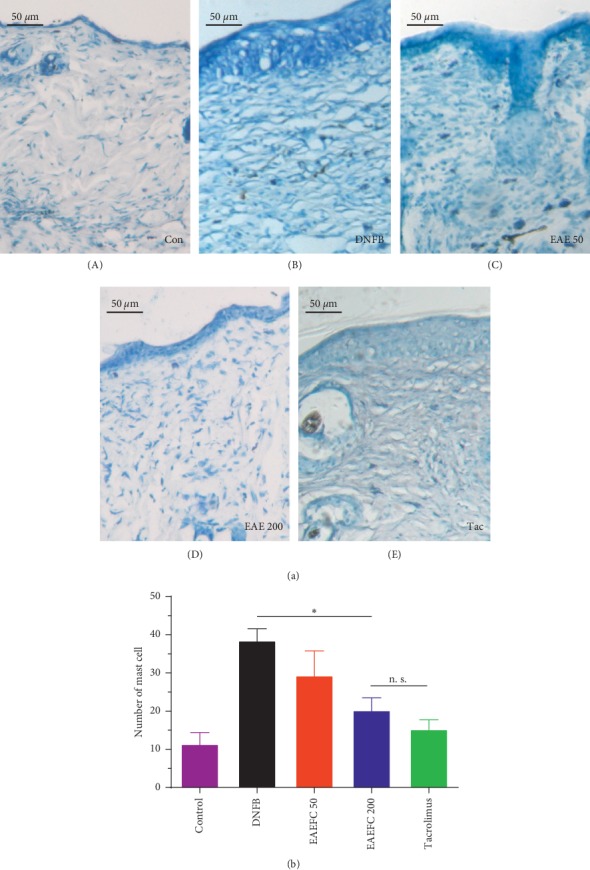
Effect of EAEFC treatment on DNFB-induced mast cell infiltration in C57BL/6 mice. (a) The TB-stained skin of mice from the (A) control group, (B) DNFB-treated group, (C) EAEFC50 group, (D) EAEFC200 group, and (E) tacrolimus-treated group on day 22. (b) Data are presented as the mean ± SEM (*N* = 4). ^*∗*^: Significant difference between the EAEFC200 and DNFB-treated groups at *P* < 0.05. n s.: No significant difference between the EAEFC200 and tacrolimus-treated groups.

The epidermal hyperplasia and the number of dermal inflammatory cells, particularly mast cells, evidently increased in the injured skin of C57BL/6 mice with DNFB-induced AD (Figure 6(a)). On day 21, the skins of DNFB-treated mice were significantly thicker than the control mice. Treatment with EAEFC50, EAEFC200, and tacrolimus markedly attenuated the DNFB-induced increase in skin thickness (Figure 6(a)). On day 21, the skin thickness in EAEFC200 mice had markedly decreased compared to DNFB-treated mice (115.7 ± 4.9 vs. 41.2 ± 4.8, *N* = 4, ^*∗∗∗*^*P* < 0.001) (Figure 6(b)). Furthermore, on day 21, the number of mast cells in the DNFB-treated group significantly increased compared to the control group, where only a few mast cells were detected (Figure 7(a)). Treatment with EAEFC50, EAEFC200, and tacrolimus reduced the DNFB-induced increase in the number of mast cells (Figure 7(a)). On day 21, the EAEFC200 group had a significantly small number of mast cells compared to the DNFB-treated group (20.3 ± 3.3 vs. 38.5 ± 3.1, *N* = 4, ^*∗*^*P* < 0.05) (Figure 7(b)). By contrast, there was no significant difference between the EAEFC and tacrolimus-treated groups (Figures 6 and 7). These results suggest that EAEFC recovered the damaged skin barrier in C57BL/6 mice with DNFB-induced AD.

### 3.6. EAEFC Alleviated the DNFB-Induced Increase of Scratching Behavior in C57BL/6 Mice

**Figure 8:**
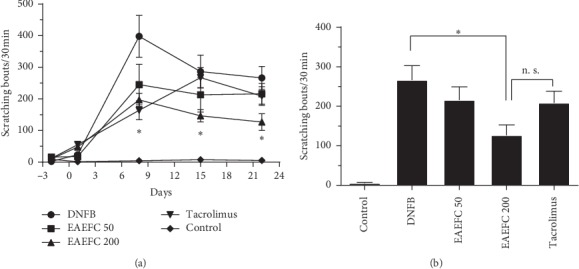
Effect of EAEFC on DNFB-induced chronic scratching behavior in C57BL/6 mice. (a) The number of scratching behavior was recording for 30 minutes on day −2, 1, 8, 15 and 22. (b) The number of scratching behavior at the last recording. Data represent the mean ± SEM (*n* = 8). ^*∗*^: EAEFC200 group *vs* DNFB-treated group, significant difference, *P* < 0.05. n. s.: EAEFC200 group *vs* tacrolimus group, no significant difference.

**Figure 9:**
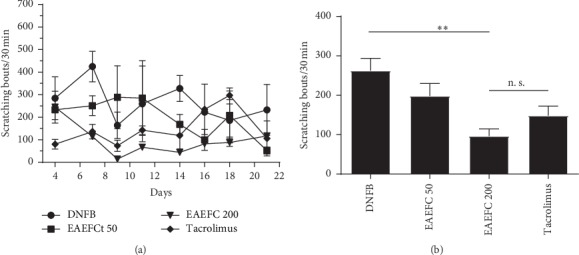
Effect of EAEFC treatment on DNFB-induced acute scratching behavior in C57BL/6 mice. (a) The number of scratching bouts was measured for 30 min after DNFB application on the day of sensitization. (b) The number of scratching bouts in all acute scratching recording. Data are presented as the mean ± SEM (*N* = 4). ^*∗∗*^: Significant difference between the EAEFC200 and DNFB-treated groups at *P* < 0.01. n. s.: No significant difference between the EAEFC200 and tacrolimus-treated groups.

The antipruritic effect of EAEFC was assessed by analyzing the spontaneous scratching behavior in mice with DNFB-induced AD. We recorded the chronic spontaneous scratching bouts per week or the acute scratching bouts immediately after DNFB sensitization. On day 8, the number of scratching bouts markedly increased in the DNFB-treated group compared to the control group (398.0 ± 66.2 vs. 4.4 ± 1.2, *N* = 8). By contrast, the EAEFC200 group had significantly reduced number of scratching bouts compared to the DNFB-treated group (197.0 ± 21.1 vs. 398.0 ± 66.2, *N* = 8, ^*∗*^*P* < 0.05) (Figure 8(a)). In addition, the antipruritic effect of EAEFC lasted until the end of the experiment. On day 22, there was a significant difference between the EAEFC200 and DNFB-treated groups (126.8 ± 26.4 vs. 266.5 ± 36.4, *N* = 8, ^*∗*^*P* < 0.05) (Figure 8(b)). Contrastingly, no significant difference was observed between the EAEFC- and tacrolimus-treated groups. DNFB-induced acute scratching desensitization was also a promising approach to demonstrate the antipruritus effect of EAEFC. To assess the effect of long-term EAEFC administration on acute scratching desensitization, the scratching behavior of mice was recorded 10 min after DNFB application. Results revealed that EAEFC can inhibit the DNFB-induced acute pruritus in mice. The number of scratching bouts in the EAEFC200 group dropped compared to the DNFB-treated group (96 ± 18 vs. 262.6 ± 31.2, *N* = 4, ^*∗*^*P* < 0.05) (Figure 9). However, there was no significant difference between the EAEFC- and tacrolimus-treated groups. These results indicate that EAEFC inhibited the DNFB-induced scratching in C57BL/6 mice.

### 3.7. EAEFC Alleviated the DNFB-Induced Increase of Serum IgE Level in C57BL/6 Mice

**Figure 10:**
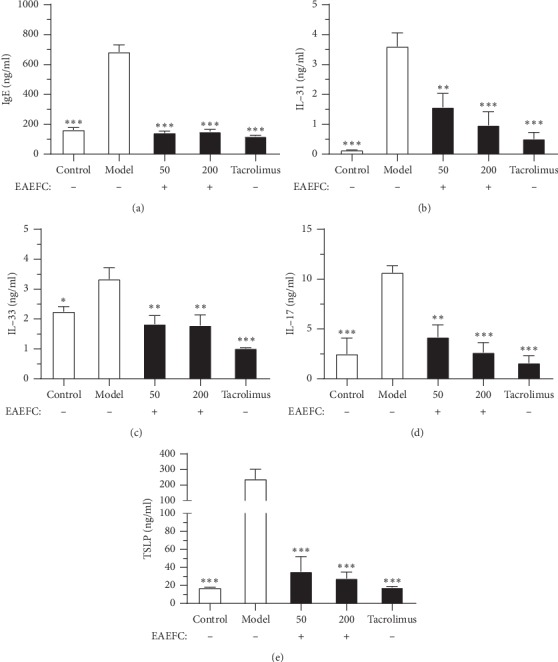
Effect of EAEFC treatment on the levels of DNFB-induced IgE and itch-related cytokines in C57BL/6 mice. The serum (a) IgE, (b) IL-4, (c) IL-33, (d) IL-17, (e) TSLP, and (f) IL-31 levels were determined using ELISA. Data are presented as the mean ± SEM (*N* = 5–8). Significant difference between each group and DNFB-treated group at ^*∗*^: *P* < 0.05, ^*∗∗*^: *P* < 0.01, and ^*∗∗∗*^: *P* < 0.001.

Hyperproduction of serum IgE is an important characteristic of AD. To further investigate the association between the EAEFC-alleviated skin lesions and serum IgE levels, we performed ELISA to compare the IgE levels between the control and treated groups. As shown in Figure 10(a), the serum IgE levels in the DNFB-treated group were significantly elevated compared to the control group (683.1 ± 47.4 vs. 162.1 ± 15.7 ng·mL^−1^, ^*∗∗∗*^*P* < 0.001). However, the serum IgE levels of EAEFC50 (142.4 ± 11.5 vs. 683.1 ± 47.4 ng·mL^−1^, ^*∗∗∗*^*P* < 0.001), EAEFC200 (152.1 ± 12.9 vs. 683.1 ± 47.4 ng·mL^−1^, ^*∗∗∗*^*P* < 0.001), and tacrolimus-treated (120.4 ± 5.5 vs. 683.1 ± 47.4 ng·mL^−1^, ^*∗∗∗*^*P* < 0.001) groups significantly decreased compared to the DNFB group. The effect of the EAEFC200 treatment is similar to the tacrolimus.

### 3.8. EAEFC Alleviated the DNFB-Induced Increase of Pruritogenic Cytokines in C57BL/6 Mice

It has been reported that increased levels of various cytokines, such as IL-17, IL-33, IL-31, and TSLP, directly correlate to scratching behavior. To further investigate the association between the EAEFC-inhibited scratching behavior and itch-related cytokines levels, we examined the serum IL-17, IL-33, IL-31, and TSLP levels in all groups using ELISA. As shown in Figure 10, the IL-17 (10.7 ± 0.7 vs. 2.5 ± 1.6 ng·mL^−1^, ^*∗∗∗*^*P* < 0.001), IL-33 (3.3 ± 0.4 vs. 2.2 ± 0.2 ng·mL^−1^, ^*∗*^*P* < 0.05), IL-31 (3.6 ± 0.4 vs. 0.1.1 ± 0.0 ng·mL^−1^, ^*∗∗∗*^*P* < 0.001), and TSLP (238.3 ± 63.7 vs. 17.5 ± 0.6 ng·mL^−1^, ^*∗∗∗*^*P* < 0.001) levels were significantly elevated in the DNFB-treated group compared to the control group. However, the serum IL-17, IL-33, IL-31, and TSLP levels in the EAEFC50, EAEFC200, and tacrolimus-treated groups significantly decreased compared to the DNFB-treated group. The IL-17, IL-33, IL-31, and TSLP levels in the EAEFC200 group were not significantly different compared to the tacrolimus-treated group. These results indicate that EAEFC alleviated the DNFB-induced increase of IL-17, IL-33, IL-31, and TSLP levels in C57BL/6 mice.

### 3.9. Discussion

Chronic itch, a common and an unpleasant symptom of AD, was induced by multiple complex factors and systems, including the nervous and immune systems [9, 37]. The prevalence of AD in American children that are 5, 9, and 15 years old was 14% to 15% [38]. Furthermore, the prevalence of pruritus due to AD ranges from 6.1% to 22.3% in western countries [39]. Interestingly, traditional Chinese medicinal herbs, such as *Fructus cnidii*, can be used for the treatment of itching in AD; however, the antipruritic mechanism of this herb is not well understood [40]. In the present study, we demonstrated that EAEFC attenuated the DNFB-induced AD-like symptoms in mice by topical application to the diseased skin. The DNFB-induced increase in skin lesion severity, dermatitis score, epidermal hyperplasia, mast cell infiltration, scratching bouts, and serum IgE levels were significantly alleviated by the EAEFC treatment. Furthermore, EAEFC application can reduce the levels of itch-related cytokines, specifically IL-17, IL-33, TSLP, and IL-31. In addition, the effect of EAEFC200 is better than EAEFC50 in attenuating the DNFB-induced AD-like symptoms. In traditional Chinese medicine, the dose of *Fructus cnidii* for a 70 kg person is 30 g·day^−1^; here, 200 mg·kg^−1^ and 50 mg·kg^−1^ of EAEFC were the safe dosages in mice. These results suggest that EAEFC has a functional role in the treatment of pruritus caused by AD.

Osthole is one of the major compounds in *Fructus cnidii* [24]. In our study, after HPLC analysis with the standard (Figure 3), it was suggested that the peak at 9.39 was the compound osthole, which are presented in the EAEFC.

Skin barrier dysfunction and amplified immune responses are well-known features of AD [41]. Skin barrier disruption in AD promotes the release of Th2 cytokines and chemokines, such as TSLP, IL-25, CCL17, CCL22, and CCL5 [42–44]. In addition, increased nerve fiber density and decreased threshold of action potential in the skin barrier were reported [11, 45]. Hence, skin barrier dysfunction and inflammation may be involved in the development of AD-induced pruritus. Breaking the vicious itch-scratching cycle and recovering the damaged skin barrier are the most commonly used treatments for AD [2, 46]. Clinical studies have revealed that immediate moisturization of the damaged skin barrier can reduce the risk of developing AD in children [47]. The H&E and TB staining analyses revealed that the skin exhibited hyperkeratosis, thick epidermis, and mast cell infiltration after repeated exposure to DNFB (Figures 6 and 7). However, we found that EAEFC treatment alleviated the skin barrier disruption and reduced the inflammatory cell infiltration in DNFB-treated mice, strongly suggesting that EAEFC may be a potential candidate for the treatment of AD-induced inflammation and pruritus (Figures 4 and 5).

Furthermore, inflammation due to AD drives the immune system to deviate towards the Th2 axis and releases TSLP and IL13, consequently triggering the B cells to produce IgE. The blood IgE level in AD patients elevated to approximately 80%, which is part of the 20 secondary criteria for diagnosing AD [48, 49]. In addition, skin barrier dysfunction and inflammation trigger the degranulation of mast cells, which contributes to the release of pruritogens containing histamine [50]. IgE can also interact with the high-affinity IgE receptor (FceRI) on mast cells and induce the cascade of inflammatory responses. Therefore, the histochemistry and ELISA were implemented to clarify the role of EAEFC in alleviating serum IgE. We discovered that serum IgE and mast cell infiltration levels were higher in the DNFB-treated group compared to the control group (Figures 7(b) and 10(a)). Moreover, the administration of EAEFC clearly reduced the IgE production and mast cell infiltration. However, studies on ear swelling have reported that 70% ethanol extract from *Fructus cnidii* has an inhibitory effect in the DNFB- and picryl chloride-induced allergy in ICR mice [30]. These results demonstrate that EAEFC has an inhibitory effect on the inflammation due to AD, possibly by decreasing the IgE production and mast cell infiltration.

Scratching behavior is an obvious effect of AD; therefore, reduced scratching is a criterion for antipruritic treatment. In C57BL/6 mice with DNFB-induced AD-like symptoms, the scratching behavior markedly increased compared to the control. However, EAEFC treatment attenuated the DNFB-induced scratching behavior. Interestingly, we found that long-term administration of EAEFC (from day 0 to 21) decreased the DNFB sensitization and acute scratching behavior. These results indicate that EAEFC has an inhibitory effect on the development of chronic and acute itch. Previous research reported that the osthole in EAEFC inhibited histamine-dependent itch through TRPV1 or TRPV3 [27, 29]. In this study, EAEFC may have desensitized the mice to DNFB; however, the mechanism of EAEFC desensitization requires further investigation.

In addition, the increased levels of itch-related cytokines are a cause of itch and they correlated with AD through epithelial stress, immune, and nervous system response [46]. TSLP, an epithelial-derived cytokine released from keratinocytes due to skin barrier dysfunction, injected intradermally into mice resulted in scratching through the activation of the itch-generating sensory neurons [18]. Our results showed that EAEFC application distinctly reduced the TSLP level in the EAEFC-treated group. The decrease in TSLP level may be associated with the effects of EAEFC in mice. IL-33, which is also an epithelial-derived cytokine, and its receptor antibodies can reduce the scratching behavior in a mouse model with poison ivy-induced allergic contact dermatitis [17]. Similarly, IL-33 also decreased after the EAEFC treatment in this study. Moreover, IL-33 and TSLP drive Th2 cells into the skin and subsequently release the type 2 cytokines IL-4, IL-5, and IL-13 [51]. However, IL-4 or IL-13 did not induce acute scratching behavior in a murine model by direct subcutaneous injection into the cheek [12]. IL-31, another Th2 cell-associated cytokine, is a pruritogen that mediate itch development by directly exciting the sensory neurons [23, 52]. In our study, IL-31 level increased in the DNFB-treated group compared to the control group. However, the elevated IL-31 level was reduced after the administration of EAEFC. Th17 cells and IL-17 are believed to be involved in AD pathogenesis and itch development [14, 15]. To determine the influence of EAEFC on IL-17 level in DNFB-treated mice, we compared the IL-17 levels in the EAEFC-treated group to the DNFB-treated group. Results demonstrated that the IL-17 level in the EAEFC-treated group was lower than that in the DNFB-treated group. Taken together, the results suggest that EAEFC may have attenuated the DNFB-induced itch in the mouse model by modulating the levels of itch-related cytokines.

## 4. Conclusions

In conclusion, despite the lack of a detailed molecular mechanism explaining the antipruritic activity of EAEFC, the results of our study clearly demonstrated that the topical application of EAEFC effectively alleviated the DNFB-induced AD-like symptoms in C57BL/6 mice. In particular, the antipruritic effects of EAEFC were demonstrated by the alleviation of epidermal hyperplasia, mast cell infiltration, and chronic or acute scratching behavior. Moreover, EAEFC treatment alleviated the levels of serum IgE and itch-related cytokines, specifically TSLP, IL-17, IL-33, and IL-31, which may be involved in the antipruritic activity of EAEFC. Finally, our findings suggest that *Fructus cnidii* may be an effective therapeutic agent that can potentially inhibit the itch caused by AD.

## Data Availability

The data used to support the findings of this study are included within the article.
